# Christopher Columbus’ flu was different to ours

**DOI:** 10.1073/pnas.2414921121

**Published:** 2024-10-31

**Authors:** Chika Edward Uzoigwe

**Affiliations:** ^a^Medical Sciences, Harcourt House, Sheffield S10 1DG, United Kingdom

Blackmore and Lloyd-Smith, using their instructive model (1), calculate a very low to modest probability of influenza, measles, and smallpox transmission to the Americas, during the voyages of Columbus and peers; in the 1400s to 1500s. This appears in conflict with some compelling historical evidence. Contemporaneous European and Native American accounts describe a condition similar to smallpox, causing pandemics in Mexico in 1520, 1545, and 1576, leading to the loss of up to 25 million lives (2). Aztec illustrations from the early/mid-1500s, in the *Florentine Codex* for example, depict features strongly resembling smallpox (3). Similarly, written accounts suggest that the Americas suffered as part of a global Influenza pandemic around 1557 that lagged behind that observed in Europe by weeks to months (4). The same applies to measles in the early 1500s (5). Granted retrospective diagnoses can be inaccurate, however, measles and smallpox are very distinctive.

The question arises as to why the probabilistic calculations seem to challenge historical evidence. The authors base their model on the infective parameters of contemporary strains. The pathogenic behavior and/or zoonotic predilection of the influenza, smallpox, and measles viruses of the 1400s to 1500s likely differ from that of the 20th–21st-century strains. Given viral replication rates, 500 y of viral evolution is unfathomable. Over this time, half a millennium of immunological warfare has raged. Further given the impact of dietary restriction on immunity, host aggressiveness, or permissiveness to viral invasion/colonization remains unclear. This is compounded by the fact that early transatlantic crews were exclusively young men. The natural histories of infection differ between men and women, for a number of microbiota (6). This effect, possibly mediated by testosterone, means that population mean pathogen parameters do not necessarily apply to a cohort of young men (6).

Zoonotic transmission and the animal pool are important factors overlooked. Transatlantic voyages conveyed large volumes of species; that are equally viral hosts, to the Americas, including livestock, birds, and rodents (7). This is relevant for influenza but also for measles and variola (smallpox) virus. Influenza far predates our species, potentially arising up to 500 Mya (8). Measles and variola viruses are recent species, evolved specifically for human hosts from zoonotic ancestors. Measles virus originated in 600s BC, from rinderpest virus which affects cattle (9). Measles variants consistently crossed the species barrier, causing concurrent human-bovine epidemics into the Middle Ages (4).

Variola virus originates from an ancestor with a rodent host (10, 11). Genomic work shows that 20th-century strains emerged only around the 1600s; earlier strains are now extinct. Medieval clades show such heterogeneity that the pathogenic behavior of such strains is un-interpolable from the latest strains (5, 6).

Strict viral *Homo*-topism could be a recent phenotype arising from exclusive human conurbation without animal cohabitation. Strains with the highest avidity for human antigens and most adept at evading human immunity would thrive over those with less specificity. However, in milieux where human–human engagement occurs in comparable frequency to human–animal/human–human–animal, greater versatility would facilitate dissemination.

Incorporation of the factors raised herein may better align historical consensus with mathematical models.
